# Coexistence of Three Dominant Bacterial Symbionts in a Social Aphid and Implications for Ecological Adaptation

**DOI:** 10.3390/insects12050416

**Published:** 2021-05-06

**Authors:** Qian Liu, Hui Zhang, Lingda Zeng, Yuhua Yu, Xiaolan Lin, Xiaolei Huang

**Affiliations:** State Key Laboratory of Ecological Pest Control for Fujian and Taiwan Crops, College of Plant Protection, Fujian Agriculture and Forestry University, Fuzhou 350002, China; liuqian9502@163.com (Q.L.); zhanghui1903@163.com (H.Z.); lingdazeng@126.com (L.Z.); yuhuaakx@foxmail.com (Y.Y.); linxl@fafu.edu.cn (X.L.)

**Keywords:** *Buchnera*, *Pectobacterium*, *Wolbachia*, endosymbiont, social aphid, ecological function

## Abstract

**Simple Summary:**

Most insects are associated with a variety of symbionts that play a crucial role in insect life history. Symbiosis of aphids and their symbionts is a good model system to study insect–symbiont interactions. *Pseudoregma bambucicola* is a typical social aphid that lives parthenogenetically throughout the year on bamboos in subtropical areas, and it is the only aphid that exclusively feeds on the hard stalks of bamboo. In this study, we surveyed the symbiotic bacterial community associated with *P. bambucicola*. Our results showed that the diversity of *P. bambucicola* microbiome was low, but three symbionts, namely the primary endosymbiont *Buchnera* and two secondary symbionts (*Pectobacterium* and *Wolbachia*), were stable coexisting with a high infection rate. Combined with the biology of *P. bambucicola*, we speculate that *Pectobacterium* may help *P. bambucicola* feed on the stalks of bamboo, and *Wolbachia* may regulate the loss of sexual reproduction or has a nutritional role in *P. bambucicola*. These findings will advance our knowledge of the microbiomes of social aphids and set the foundation for further studies on the functional roles of *P. bambucicola* symbionts.

**Abstract:**

Aphids are associated with an array of symbionts that have diverse ecological and evolutionary effects on their hosts. To date, symbiont communities of most aphid species are still poorly characterized, especially for the social aphids. In this study, high-throughput 16S rDNA amplicon sequencing was used to assess the bacterial communities of the social aphid *Pseudoregma bambucicola*, and the differences in bacterial diversity with respect to ant attendance and time series were also assessed. We found that the diversity of symbionts in *P. bambucicola* was low and three dominant symbionts (*Buchnera*, *Pectobacterium* and *Wolbachia*) were stably coexisting. *Pectobacterium* may help *P. bambucicola* feed on the hard bamboo stems, and genetic distance analysis suggests that the *Pectobacterium* in *P. bambucicola* may be a new symbiont species. *Wolbachia* may be associated with the transition of reproduction mode or has a nutritional role in *P. bambucicola*. Statistical tests on the diversity of bacterial communities in *P. bambucicola* suggest that aphid populations attended by ants usually have a significantly higher evenness than populations without ant attendance but there was no significant difference among aphid populations from different seasons.

## 1. Introduction

Associations between bacteria and insects are widespread in nature. Bacterial symbionts associated with insects may have diverse ecological and evolutionary effects on their hosts [1,2,3,4]. For examples, *Wolbachia* are widespread in insects as master manipulators of host biology [1], and *Buchnera* and *Wigglesworthia* provide essential nutrients to their host aphids and tsetses, respectively [5,6]. The microbiota of the insect gut often plays an important role in regulating the host’s metabolism [7].

Phloem sap-feeding aphids (Hemiptera: Aphididae) feed on a wide variety of host plant species, they represent serious pests and act as vectors of phytopathogenic viruses and bacteria [8,9]. The symbiotic relationship that aphids form with many beneficial intracellular bacteria is believed to be one of the major reasons for their ecological success [10,11]. The symbiont communities associated with aphids are diverse and they form one of the most studied model systems for investigation of bacteria–insect symbiosis. Nearly all aphids are infected with the obligate endosymbiont *Buchnera aphidicola* [6], which are localized within specific cells, i.e., the bacteriocytes, and are strictly vertically transmitted from mother to offspring [6]. Aphid hosts can get essential amino acids and vitamins from *Buchnera*, which are scanty in the phloem sap diet [6,12].

In addition to *Buchnera*, aphids may harbor one or several secondary or facultative symbionts [13]. Unlike *Buchnera*, those diversified bacterial lineages are horizontally transferred within and across host species in addition to vertical transmission, and they usually occur in a fraction of host populations. Although facultative symbionts may be non-essential for host survival, they provide ecological benefits for hosts, such as host plant use [2], defense against pathogens and natural enemies [14,15], body color regulation [16], heat tolerance [17], and manipulation of host reproduction [18]. The positive relation between secondary endosymbionts and plant utilization of aphid hosts has been reported by some studies [2,19,20]. In pea aphid populations in Japan, *Regiella insecticola* infection may improve the fitness of the pea aphids specifically on white clover [2]. The effects on plant-specific fitness of infection of this secondary endosymbiont can also occur in pea aphid populations in Californian and France [21,22]. It has been reported that one symbiont occurs more frequently in unrelated aphid species that feed on certain plant genera, and aphid species that attack multiple plants often carry different symbiont complements [23].

Studies have shown that the prevalence of some symbionts in aphids is related to complex factors, such as the aphid species [24,25], the host plant association [2,26], climatic factors [26,27], and even ant attendance [28]. The diversity and prevalence of symbionts often vary between host species and populations [24,29,30], and it is possible that the same species may have different symbiotic communities at different times. Therefore, high-resolution time series data should provide a much more precise understanding of the aphid-symbiont relationship and help discriminate between bacteria that are prevalent with stable functioning in one species and those that only exist in some individuals. The mutualistic relationships between certain ants and aphid species are well known. Fischer et al. (2015) reported that microorganisms living in aphid honeydew may be able to alter emissions of volatile organic compounds (VOCs) and significantly mediate ant partner attraction [31], suggesting a possible relation between aphid symbiont composition and stability of aphid–ant mutualism.

Although relationships between aphids and symbiotic bacteria have received considerable attention, most previous studies have focused on the model pea aphid, *Acyrthosiphon pisum* [13]. The knowledge of diversity and function of symbiotic bacteria of other aphid species remains limited. Our current study focuses on a social aphid *Pseudoregma bambucicola* (Takahashi), which belongs to the tribe Cerataphidini and is mainly distributed in subtropical areas of Asia. This species has a complex life history and interesting biological traits [32,33]. In specific high-altitude areas of Taiwan Island, it can alternate between sexual and asexual generations on primary host plants, *Styrax species*, and secondary host plants, usually *Bambusa* bamboos, respectively. However, throughout most of its distribution, including areas such as southern China and southern Japan (the Ryukyus, Kyushu and Shikoku), *P. bambucicola* reproduces parthenogenetically all year around and forms dense colonies on bamboo stems [34]. Like other social insects, *P. bambucicola* produces soldier individuals to protect clones from natural enemies [34]. Small clones of *P. bambucicola* on bamboos are often attended by ants and with few soldiers, while most large colonies produce numerous soldiers and do not maintain an association with ants [35]. The attraction of ants may be an alternative way that aphids use to protect themselves from natural enemies. For *P. bambucicola* populations, it will be interesting to investigate whether the composition of symbiotic bacteria is influenced by ant attendance or not. Moreover, based on accumulated evidence [34] and our long-term field observations, *P. bambucicola* is probably the most specialized aphid species feeding on the hard stem of bamboo, while the other aphid species using bamboos as host plants usually feed on leaves or other softer parts [34]. We may therefore hypothesize that in *P. bambucicola* there should be a relation between specific symbiotic bacteria and utilization of the hard stem of bamboo. Although *P. bambucicola* can be a good choice to study ecological complexity and symbiotic association, most previous studies of this species have been conducted on field ecology [36] and behavior [32,35], but no study has specifically aimed to analyze the diversity of symbionts within this species.

The rapid development of high-throughput sequencing has made it easier to detect the entire bacterial communities of insects. In contrast, although the traditional PCR-based approach has been used to detect specific symbiotic bacteria within hosts, it may omit many other non-target cohabiting bacteria. In this study, the 16S rRNA gene amplicon sequencing was used to assess the entire symbiotic bacterial community of the social aphid *P. bambucicola*. We also examined whether the symbiotic bacterial composition can be stable along a time series (in different seasons) and whether it can be influenced by ant attendance or not.

## 2. Materials and Methods

### 2.1. Aphid Collection

Aphid samples from twenty-eight *P. bambucicola* populations on *Bambusa* bamboos in subtropical areas of southern China were collected from August 2015 to January 2018. Detailed sampling information is listed in Table 1. In order to examine temporal dynamics of the symbiotic bacterial community of *P. bambucicola*, the samples from Fujian province were assigned into four groups based on seasons of a year (‘Time groups’ in Table 1). These samples were also divided into two groups based on whether the collected aphid clones were attended by ants or not (‘Ant groups’ in Table 1). Each sample comprised of multiple individuals collected from one same aphid colony from a single bamboo stem. The number of aphids contained in each sample ranged from a few dozen to a few hundred, depending on the size of the clone. The specimens were kept in 95% ethanol and store at −20 °C after collection.

### 2.2. DNA Extraction

An apterous adult aphid from each sample was washed three times in ultrapure water. Total genomic DNA was extracted from whole individuals with the DNeasy Blood & Tissue kit (QIAGEN) according to the manufacturer’s instructions. DNA extraction was carried out in an ultra-clean workbench to avoid contamination of environmental DNA. The bacterial universal primers 8F (5′-AGAGTTTGATCCTGGCTCAG-3′) and 1492R (5′-GGTTACCTTGTTACGACTT-3′) [37] were used to verify the success and quality of the DNA extractions. To assure the accuracy of results, sterile deionized water was used as a negative control. PCR amplifications were performed in 25 μL reactions containing 1 μL of DNA, 2.5μL 10× LA PCR buffer II (Mg^2+^ plus), 0.5 μL dNTP mixture (2.5 mM each), 0.5 μL of each primer (10 μM), 0.5 μL of TaKaRa LA Taq (5 U/µL) (TaKaRa Bio Inc., Otsu, Japan), and 19.5 μL water. Amplification was performed in ProFlex^TM^ Base (Applied Biosystems, Inc., Waltham, United States), using the following cycling conditions: 94 °C for 4 min, followed by 30 cycles of 30 s at 94 °C, 40 s at 65 °C, 90 s at 72 °C, and a final extension of 10 min at 72 °C. The PCR products were detected on 1% agarose gels, and the positive samples with a bright band of about 1500 bp were kept at −20 °C until 16S library preparation. The negative controls had no bands.

### 2.3. 16S rRNA Gene Amplification and Sequencing

The primers 338F (5′-ACTCCTACGGGAGGCAGCA-3′) and 806R (5′-GGACTACHVGGGTWTCTAAT-3′) [38] were used to amplify the V3 and V4 regions of the 16S ribosomal RNA (rRNA) gene with 95 °C for 5 min (1 cycle), 95 °C for 30 s, 50 °C for 30 s, 72 °C for 40 s (25 cycles), followed by 72 °C for 7 min. The PCR products were purified, quantified, and homogenized to form a sequencing library. The established library was checked for quality first, and paired-end sequencing of the 16S rDNA was conducted on Illumina HiSeq 2500 with 2 × 250 bp reads (Illumina, Inc., San Diego, CA, USA) at Biomarker Bioinformatics Technology, Co., Ltd. (Beijing, China).

### 2.4. OTU Clustering and Taxonomic Assignment

According to the overlap relation between paired-end reads, the PE reads were merged into single, longer raw tags using FLASH v1.2.11 [39]. Raw tags were further quality trimmed to obtain clean tags using Trimmomatic v0.33 [40] ensuring > 20 quality scores on a sliding window of 50 bp. The chimera sequences were identified and removed using the UCHIME v8.1 [41]. Sequences with ≥ 97% similarity were assigned to the same operational taxonomic units (OTUs) using USEARCH v10.0 [42]. OTUs with a number of sequences < 0.005% of the total number of sequences were discard [43]. Representative sequences from each OTU were screened for further annotation. For each representative sequence, the Silva [44] was used with the RDP classifier v2.2 [45] to annotate taxonomic information. To further confirm the taxonomic assignment, the representative OTU sequences were compared to the sequences in GenBank using BLAST.

The results of taxonomic assignment of two dominant bacteria, *Pectobacterium* and *Wolbachia*, were further confirmed by reconstructing phylogenetic trees. The representative sequence of the most abundant OTU of *Pectobacterium* (OTU3) was extracted. Representative 16S rDNA sequences of *Pectobacterium*, *Dickeya*, and *Erwinia* were downloaded from GenBank. Sequences were aligned using MAFFT v 7.427 [46] with removal of sequences not in the same regions using MEGA 7.0 [47], and *Escherichia coli* (GenBank accession number, NR_024570.1) was chosen as the outgroup. A total of 10 *Pectobacterium* sequences, 11 *Dickeya* sequences, and 4 *Erwinia* sequences of 434 bp length were used to construct a maximum likelihood (ML) phylogenetic tree. In addition, the representative OTU sequence of *Wolbachia* (OTU5) was also extracted, and representative 16S sequences of *Wolbachia* were downloaded from GenBank. All sequences of *Wolbachia* were treated with the same steps as *Pectobacterium*, and *Rickettsia endosymbiont* (GenBank accession number, LN829697.2) was chosen as the outgroup. Finally, 29 *Wolbachia* sequences of 409 bp length were used to construct a ML phylogenetic tree. The phylogenetic trees of *Pectobacterium* and *Wolbachia* were reconducted by IQ-TREE v1.6.8 using the HKY+F+R2 model with 2000 SH-aLRT bootstrap replicates [48]. The pairwise genetic distances among those two sequence datasets were also calculated in MEGA 7.0 with the Kimura 2-parameter (K2P) model [49].

### 2.5. Microbiome Diversity

Species richness (Chao1 and ACE), diversity (Simpson and Shannon), and the coverage of library for each aphid sample were calculated in Mothur v.1.30 [50]. The Chao1 and ACE indices indicate species richness (number of microbial species). The Shannon and Simpson indices measure species diversity by taking into consideration species abundance and species evenness in the sample community. In the case of same species richness, the larger Shannon index and smaller Simpson index indicate the larger evenness of each species and the greater diversity of community [51]. These different indices for the ‘Ant groups’ and ‘Time groups’ were then compared, respectively, using one-way ANOVA with the IBM Statistical Package for the Social Sciences (SPSS) version 24.0 (Chicago, IL, USA).

## 3. Results

### 3.1. Library Basic Statistics

Using Illumina’s stringent quality control (QC), we obtained a total of 2,150,533 Clean tags. The mean number of Clean tags generated for each sample was 76,805 (SD = 19,897.854). After sequence filtering steps, we obtained 2,093,909 reads (Table S1). The assembly of paired sequences resulted in consensus sequences with an average length of 431 bp.

### 3.2. OTU Clustering and Taxonomic Assignment

High-quality reads were clustered using > 97% sequence similarity into 23 microbial OTUs (Table 2); the number of OTU in each sample is presented in Table S2. Table S3 shows the details of OTUs distribution in all samples, the raw sequences have been submitted to the NCBI Sequence Read Archive (accession number: PRJNA634633).

**Figure 1:**
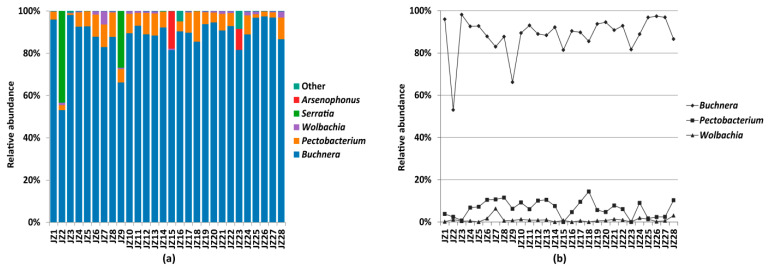
(**a**) Taxonomic composition and relative abundance of symbiotic bacteria at genus level for all *Pseudoregma bambucicola* samples; (**b**) relative abundance of the three dominant symbionts.

Of the total reads for 28 *P. bambucicola* samples, 99.94% OTUs were classified as Proteobacteria and most bacteria were from the Enterobacteriaceae (98.45% of the total reads). The obligate endosymbiont *Buchnera* was the most abundant taxon and was detected in all aphid samples (89.07% of the total reads) and was represented by two OTUs (OTU1, 56). Other taxa with relatively high abundance included *Pectobacterium* (6.56%, OTU3, 9, 104, 322, 427), *Wolbachia* (0.97%, OTU5), *Serratia* (1.72%, OTU2), and *Arsenophonus* (1.09%, OTU4). It is worth noting that *Pectobacterium* was detected in 26 of 28 aphid samples, and its relative abundance fluctuated between 0.73% and 14.41% in different samples. A similar situation occurred for *Wolbachia* which was detected in 23 of 28 aphid samples, with relative abundance fluctuating between 0.14% and 6.33%. Whilst another well-known insect symbiont, *Serratia*, was detected in 13 samples, its relative abundance was ≤0.01% (reads number < 5) in 11 of the 13 samples. *Arsenophonus* sequences were represented in six aphid samples, but four of which had a relative abundance ≤ 0.01% (reads number < 12). Table 3 shows the detailed information of the top five most abundant bacteria. The relative abundance of the top five genera in 28 samples are illustrated in Figure 1a, while the relative abundance of three dominant symbionts in all samples are shown in Figure 1b.

**Figure 2:**
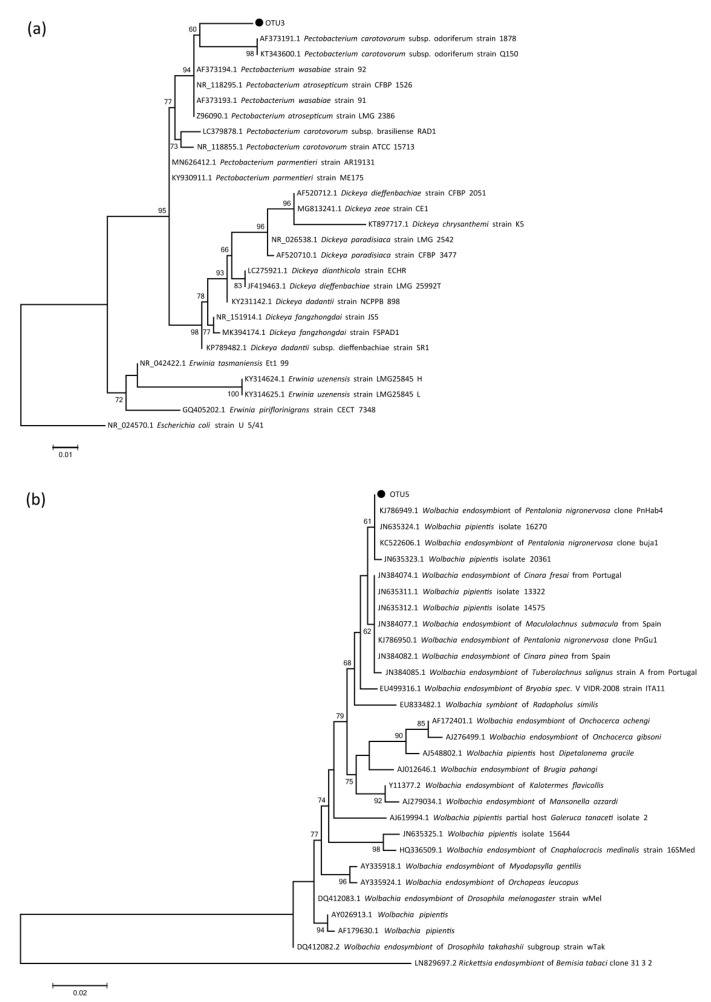
The maximum likelihood phylogenic tree of (**a**) *Pectobacterium* and (**b**) *Wolbachia* based on 16s rDNA V3–V4 region. OTU3 and OTU5 represent the main OTU of *Pectobacterium* and *Wolbachia* from all *Pseudoregma bambucicola* samples, respectively. Numbers above the branches indicate the bootstrap support values, for which lower than 50 are not shown.

In the *Pectobacterium* phylogenetic tree (Figure 2a), OTU3 and other *Pectobacterium* species clustered together, while *Dickeya* and *Erwinia* formed separate clades, respectively. The genetic distances between OTU3 and all other *Pectobacterium* sequences ranged from 2.1% to 3.9%, which indicated that the *Pectobacterium* symbiont in *P. bambucicola* may represent a new bacterial species of this genus. As the *Wolbachia* phylogenetic tree (Figure 2b) shows, with a low support OTU5 and several other *Wolbachia* strains from aphids clustered together, and *Wolbachia* strains of other arthropods and nematodes separated from this clade. The genetic distances between OTU5 and all other *Wolbachia* sequences ranged from 0 to 3.8%, while the V3–V4 region of 16s rDNA sequence of *Wolbachia* from *P. bambucicola* was identical with three *Wolbachia* sequences from other aphids, indicating that the *Wolbachia* strain in *P. bambucicola* is a common one among aphid species.

### 3.3. Microbiome Diversity for the Ant and Time Groups

The coverage of library for each aphid sample was good, being more than 0.99 for all samples. We calculated the richness (Chao1 and ACE) and diversity (Simpson and Shannon) indices, which are shown in the Table S2. The mean Shannon diversity index was low, suggesting a low bacterial diversity in *P. bambucicola* and that each sample was dominated by only a few bacteria. The ant-attended aphid group had a significantly higher Shannon index (*F*_0.05__(1,24)_ = 12.233, *p* = 0.002) and lower Simpson index (*F*_0.05__(1,24)_ = 11.525, *p* = 0.002) than the aphid group not attended by ants, however, the Chao1 (*F*_0.05__(1,24)_ = 0.079, *p* = 0.781) and ACE (*F*_0.05__(1,24)_ = 0.002, *p* = 0.967) indices had no significant difference (Table 4). For the four time series groups, except the ACE index, there were no significant differences for the Chao1 (*F*_0.05__(3,22)_ = 1.490, *p* = 0.245), Shannon (*F*_0.05__(3,22)_ = 1.960, *p* = 0.150), and Simpson (*F*_0.05__(3,22)_ = 1.167, *p* = 0.345) indices (Table 5).

## 4. Discussion

Our study examined the symbiotic bacterial community harbored by the social aphid *P. bambucicola* and compared the microbiomes in different aphid clones of four time groups and two ant groups. Overall, 16S rDNA sequencing showed a stable coexistence of three dominant bacterial symbionts, namely the primary aphid endosymbiont *Buchnera*, the *Pectobacterium*, and the *Wolbachia*. In addition to these three symbionts, *Arsenophonus*, *Serratia*, and Orbaceae sp., previously reported to be associated with insects, were also found with relative high abundance in our study but were only limited to a few samples (Table S3). It is notable that *Arsenophonus* was only infected with two samples in which *Pectobacterium* was absent (Table 3). The genus *Arsenophonus*, a group of symbionts that kill male eggs of the wasp *Nasonia vitripennis* [52], has also been reported in other insects [53,54], but its function in many insects is far from clear.

The clones of *P. bambucicola* were stably infected by three symbiotic bacteria genera (*Buchnera*, *Pectobacterium*, and *Wolbachia*). *Buchnera*, the obligate endosymbiont of aphids, was the most abundant symbiotic bacteria of *P. bambucicola* and was detected in all samples. Fukatsu et al. (1994), using histochemical-based approaching analyses, revealed that *P. bambucicola* contain the typical intracellular symbiont *Buchnera*, rather than being replaced by a yeast-like fungus [55]. The stable coexistence of two secondary endosymbiotic bacteria is not common in aphids. For example, in spite of the most studied pea aphid containing eight to nine secondary endosymbionts, double infections with two secondary symbionts were rarely detected [56,57]. Gómez-Valero et al. (2004) found a coexistence of *Wolbachia* and a secondary symbiont (R type, *Serratia*) in the aphid *Cinara cedri* [58]. Interestingly, the coexistence of *Pectobacterium* and *Wolbachia* with high infection rate (78.57%) was detected in *P. bambucicola*. Meanwhile, compared to other aphid species with a variety of host plants, such as the pea aphid [13], the diversity of the bacterial community in *P. bambucicola* was low, likely because the habit (feeding on bamboo stems) of *P. bambucicola* is relatively simple.

High infection rates (26/28) of *Pectobacterium* have been detected in *P. bambucicola*. Such high prevalence suggests a potential functional role of *Pectobacterium* in this social aphid. The genus *Pectobacterium* are important causative agents of the soft rot disease with broad host plant ranges [59,60]. These bacteria produce a large amount of plant cell wall-degrading enzymes (PCWDEs) such as pectinases, cellulases, hemicellulases, and proteinases which makes plant tissue decay efficiently [61]. Among the over 5000 known aphid species, *P. bambucicola* is the only one that exclusively feeds on the hard stalks of bamboo, which consist of much harder cell walls than many other plants. To feed on the phloem sap of bamboo, aphid stylet must conquer the hindrance of the hard cell wall [62]. We speculate that *Pectobacterium* symbionts should help the host aphid to secrete PCWDEs, especially pectinases, to lyse and pierce the cell walls of bamboo. This may be one of the main reasons why *P. bambucicola* can adapt to the hard stalks of bamboo. In addition, our ongoing subsequent experiments of bacterial screening in different tissues of *P. bambucicola* found that *Pectobacterium* was mainly distributed in the ovary (unpublished data), indicating that *Pectobacterium* is likely a symbiotic bacterium and can vertically transmit in this aphid. It will be valuable to investigate how this symbiosis contributes to feeding adaptation of the host aphid at genomic levels for both *Pectobacterium* and *P. bambucicola*. Notably, genetic distances indicated that the *Pectobacterium* in *P. bambucicola* may be a new symbiont species and phylogenetic analysis showed that *Pectobacterium* as a symbiont in *P. bambucicola* may reflect an evolutionary transition from plant pathogen to insect symbiotic bacteria. Future localization observation such as FISH (fluorescence in situ hybridization) and comparative genomic study will also help to understand their symbiotic relationship.

*Wolbachia* is a widely distributed intracellular symbiont in arthropods and nematodes and is known as a reproductive manipulator [1]. It can alter the reproductive pattern of hosts in diverse ways, such as feminization, parthenogenesis, male killing, and sperm–egg incompatibility [1]. *Wolbachia*-induced female parthenogenesis has been documented in species such as mites, hymenopterans, and thrips [63,64,65]. In terms of aphids, a few previous PCR-based detections suggested *Wolbachia* does not have a high infection rate in aphids [66,67]. However, our current study indicates a high infection rate (23/28) of *Wolbachia* in the social aphid *P. bambucicola*. Although *Wolbachia*-induced reproductive manipulation has been demonstrated in many arthropods, this function has not been detected in aphid species. *P. bambucicola* typically live in subtropical areas and reproduce parthenogenetically, but rare records of sexual generation were reported in high altitude areas of Taiwan Island [34]. Therefore, it may be possible that this aphid has been experiencing the loss of the sexual generation in its distribution areas, and the highly infected *Wolbachia* may potentially have a role in regulating the loss of sexual reproduction in *P. bambucicola* populations. On the other hand, *Wolbachia* was demonstrated to act as a nutritional mutualistic bacterium in other insects, such as the aphid *Pentalonia nigronervosa* [68] and the bedbug *Cimex lectularius* [3]. Whether *Wolbachia* has a nutritional role in *P. bambucicola* needs to be investigated by further studies based on genome analysis, microscopic observation, and functional verification experiments.

Aphid groups visited by ants had a significantly higher diversity of bacterial communities than those not attended by ants. However, Chao1 and ACE indices, having no significant difference, suggested that ant attendance had no influence on the richness, while the symbiotic microorganisms of aphids attended by ants had higher community evenness. Ant-attended *P. bambucicola* populations having higher diversity of bacterial community may indicate that ant attendance could be a selection agent for shaping different symbiotic communities, which may influence the composition of aphid honeydew and thus adjust its attraction to ant partners. Besides, it has been demonstrated in some studies that ants can protect aphid clones from parasitoids and pathogens [69,70]. Henry et al. (2015) found that *Hamiltonella defensa* and *R. insecticola*, two protective aphid symbionts, were more likely to occur in aphid species not attended by ants [23]. Mandrioli et al. (2016) found that if aphid populations recurrently tended by ants were maintained in insectaries without ants, aphids increased the amount of *H. defensa*, making the composition of their microbiome context-dependent [28]. The associations with ants or protective symbionts may be two alternative ways that aphids can protect themselves from pathogens and nature enemies. In the field, *P. bambucicola* often encounter many kinds of natural enemies, and ants and soldier aphids alternately protect the clones from natural enemies [35]. This may be the potential reason why the number of microbial species in the *P. bambucicola* is not affected by ant attendance. In terms of the time series groups, there was no significant difference in the composition of bacterial communities, which confirms that the microbiota is dominated by three symbionts (*Buchnera*, *Pectobacterium*, *Wolbachia*) and time is not an important structuring factor for bacterial communities associated with *P. bambucicola*.

## 5. Conclusions

In this study, we reported the symbiotic bacterial community of the social aphid *P. bambucicola*. The diversity of the bacterial community in *P. bambucicola* was low and the microbiota of *P. bambucicola* was dominated by three symbiotic bacteria. In addition to the obligate endosymbiont *Buchnera*, high infection rates of *Pectobacterium* (92.9%) and *Wolbachia* (82.1%) in *P. bambucicola* may suggest a potential functional role in *P. bambucicola*. Combined with the biology of *P. bambucicola*, the symbiotic *Pectobacterium* may help *P. bambucicola* adapt its unique niche of bamboo stems, and the well-known reproductive manipulator *Wolbachia* may regulate the loss of sexual reproduction or have a nutritional role in *P. bambucicola*. The genetic distance analysis indicated that the *Pectobacterium* of *P. bambucicola* may be a new symbiont species which needs to be further validated with more data in future studies. Moreover, our study indicates that time is not an important structuring factor for bacterial communities associated with *P. bambucicola*, but the evenness of microbiota in *P. bambucicola* attended by ants is higher than that without ant attendance.

## Figures and Tables

**Table 1:** Information of *Pseudoregma bambucicola* samples used in this study.

Sample ID	Collection Dates	Ant Groups	Time Groups	Collection Localities
JZ1	5 January 2017	B	4	Fuzhou, Fujian
JZ2	8 January 2017	A	4	Fuzhou, Fujian
JZ3	18 January 2017	B	4	Xiamen, Fujian
JZ4	17 February 2017	B	4	Fuzhou, Fujian
JZ5	3 March 2017	B	1	Fuzhou, Fujian
JZ6	14 April 2017	A	1	Fuzhou, Fujian
JZ7	14 April 2017	A	1	Fuzhou, Fujian
JZ8	7 May 2017	B	1	Fuzhou, Fujian
JZ9	20 May 2017	A	1	Fuzhou, Fujian
JZ10	9 June 2017	A	2	Fuzhou, Fujian
JZ11	9 June 2017	B	2	Fuzhou, Fujian
JZ12	22 June 2017	A	2	Xiamen, Fujian
JZ13	22 June 2017	B	2	Xiamen, Fujian
JZ14	30 June 2017	B	2	Fuzhou, Fujian
JZ15	15 August 2016			Linhai, Zhejiang
JZ16	22 August 2015			Leshan, Sichuan
JZ17	22 September 2017	A	3	Fuzhou, Fujian
JZ18	26 September 2017	A	3	Fuzhou, Fujian
JZ19	20 October 2017	A	3	Fuzhou, Fujian
JZ20	20 October 2017	B	3	Fuzhou, Fujian
JZ21	4 November 2017	B	3	Fuzhou, Fujian
JZ22	4 November 2017	A	3	Fuzhou, Fujian
JZ23	10 November 2017	A	3	Fuzhou, Fujian
JZ24	10 November 2017	B	3	Fuzhou, Fujian
JZ25	6 December 2017	B	4	Fuzhou, Fujian
JZ26	22 December 2017	B	4	Fuzhou, Fujian
JZ27	17 January 2018	B	4	Fuzhou, Fujian
JZ28	17 January 2018	B	4	Fuzhou, Fujian

Note: For the ‘Ant groups,’ A represents aphid clones attended by ants, while B represents aphid clones not attended by ants. For the ‘Time groups,’ group 1 represents samples collected from the spring (March to May), while groups 2, 3, 4 represent samples from the summer (June to August), autumn (September to November) and winter (December to February), respectively.

**Table 2:** Taxonomic assignment of bacterial OTUs in *Pseudoregma bambucicola.*

OTU ID	Taxon Annotation
OTU1	*Buchnera*
OTU56	*Buchnera*
OTU3	*Pectobacterium*
OTU104	*Pectobacterium*
OTU322	*Pectobacterium*
OTU427	*Pectobacterium*
OTU9	*Pectobacterium*
OTU5	*Wolbachia*
OTU6	*Orbaceae* sp.
OTU2	*Serratia*
OTU4	*Arsenophonus*
OTU7	*Nocardioides*
OTU8	Candidatus_*Alysiosphaera*
OTU10	*Acinetobacter*
OTU11	*Escherichia-Shigella*
OTU12	*Thiothrix*
OTU13	*Sphaerotilus*
OTU14	*Nicotiana*
OTU15	uncultured_bacterium
OTU16	*Microbacterium*
OTU17	*Paracoccus*
OTU19	*Fusobacterium*
OTU24	*Bacteroides*

**Table 3:** Reads and relative abundance of symbiotic bacteria in each sample, emphasizing the major five bacteria.

Sample ID	*Buchnera*	*Pectobacterium*	*Wolbachia*	*Serratia*	*Arsenophonus*	Other
JZ1	41,346 (95.96%)	1633 (3.79%)	59 (0.14%)	0	0	47 (0.11%)
JZ2	25,062 (53.07%)	1139 (2.41%)	541 (1.15%)	20,472 (43.35%)	0	8 (0.02%)
JZ3	34,163 (98.16%)	253 (0.73%)	138 (0.40%)	2 (0.01%)	0	249 (0.72%)
JZ4	67,789 (92.58%)	4963 (6.78%)	376 (0.51%)	4 (0.01%)	0	88 (0.12%)
JZ5	46,056 (92.75%)	3568 (7.19%)	0	0	0	30 (0.06%)
JZ6	57,971 (87.84%)	6923 (10.49%)	1075 (1.63%)	3	0	22 (0.03%)
JZ7	50,186 (82.96%)	6450 (10.66%)	3830 (6.33%)	0	0	26 (0.04%)
JZ8	57,740 (87.75%)	7604 (11.56%)	405 (0.62%)	1	0	50 (0.08%)
JZ9	38,469 (66.20%)	3637 (6.26%)	412 (0.71%)	15,569 (26.79%)	0	26 (0.04%)
JZ10	94,714 (89.45%)	9798 (9.25%)	1345 (1.27%)	1	0	22 (0.02%)
JZ11	82,091 (93.06%)	5344 (6.06%)	768 (0.87%)	1	0	11 (0.01%)
JZ12	90,991 (88.99%)	10,331 (10.10%)	857 (0.84%)	0	0	74 (0.07%)
JZ13	53,563 (88.38%)	6353 (10.48%)	635 (1.05%)	1	0	51 (0.08%)
JZ14	66,412 (92.21%)	5434 (7.54%)	0	1	1	178 (0.25%)
JZ15	67,531 (81.39%)	0	639 (0.77%)	1	14,721 (17.74%)	76 (0.09%)
JZ16	66,655 (90.39%)	3465 (4.70%)	0	0	0	3618 (4.91%)
JZ17	76,662 (89.77%)	8129 (9.52%)	493 (0.58%)	0	2	111 (0.13%)
JZ18	60,662 (85.53%)	10,221 (14.41%)	0	0	0	44 (0.06%)
JZ19	73,599 (93.77%)	4408 (5.62%)	440 (0.56%)	0	0	45 (0.06%)
JZ20	81,166 (94.56%)	4039 (4.71%)	592 (0.69%)	0	0	42 (0.05%)
JZ21	99,098 (90.82%)	8526 (7.81%)	1457 (1.34%)	0	1	37 (0.03%)
JZ22	81,033 (92.90%)	5319 (6.10%)	828 (0.95%)	0	11 (0.01%)	36 (0.04%)
JZ23	66,941 (81.64%)	1	0	0	8133 (9.92%)	6924 (8.44%)
JZ24	87,857 (88.92%)	8940 (9.05%)	1880 (1.90%)	1	0	131 (0.13%)
JZ25	101,550 (96.83%)	1745 (1.66%)	1406 (1.34%)	0	0	169 (0.16%)
JZ26	61,911 (97.40%)	1498 (2.36%)	115 (0.18%)	0	0	39 (0.06%)
JZ27	88,619 (96.90%)	2230 (2.44%)	535 (0.59%)	1	0	65 (0.07%)
JZ28	45,161 (86.60%)	5368 (10.29%)	1577 (3.02%)	0	0	43 (0.08%)

Note: Relative abundance less than 0.01% is not shown.

**Table 4:** Results of one-way ANOVA for the effect of ant attendance on bacterial community diversity of *Pseudoregma bambucicola*.

Indices	Ant Groups	*n*	M ± SE	*F*	*p*-Value
ACE	A	11	17.20 ± 0.67	0.002	0.967
	B	15	17.24 ± 0.72		
Chao1	A	11	16.09 ± 0.60	0.079	0.781
	B	15	16.32 ± 0.55		
Simpson	A	11	0.73 ± 0.04	11.525	*0.002*
	B	15	0.87 ± 0.02		
Shannon	A	11	0.57 ± 0.06	12.233	*0.002*
	B	15	0.34 ± 0.04		

Note: Group A and Group B represent aphid clones attended by ants or not, respectively. Significant *p* values (*p* < 0.05) are in italics.

**Table 5:** Results of one-way ANOVA for the effect of different seasons on bacterial community diversity of *Pseudoregma bambucicola*.

Indices	Time Groups	*n*	M ± SE	*F*	*p*-Value
ACE	1	5	15.39 ± 0.93	3.205	*0.043*
	2	5	19.68 ± 1.37		
	3	8	16.87 ± 0.76		
	4	8	17.18 ± 0.64		
Chao1	1	5	14.87 ± 0.85	1.490	0.245
	2	5	17.50 ± 1.14		
	3	8	16.31 ± 0.71		
	4	8	16.19 ± 0.55		
Simpson	1	5	0.73 ± 0.06	1.167	0.345
	2	5	0.82 ± 0.02		
	3	8	0.81 ± 0.03		
	4	8	0.85 ± 0.06		
Shannon	1	5	0.59 ± 0.09	1.960	0.150
	2	5	0.43 ± 0.03		
	3	8	0.44 ± 0.04		
	4	8	0.34 ± 0.09		

Note: Group 1 represent samples collected from the spring (March to May), while Groups 2, 3, 4 represent samples from the summer (June to August), autumn (September to November) and winter (December to February), respectively. Significant *p* values (*p* < 0.05) are in italics.

## Data Availability

The raw sequencing data are deposited in the NCBI Sequence Read Archive (SRA) database with accession number PRJNA634633.

## Supplementary Materials

The following are available online at https://www.mdpi.com/article/10.3390/insects12050416/s1, Table S1: The Illumina HiSeq results of bacterial 16S rRNA genes, Table S2: Detail information of microbiome diversity indices of 28 *Pseudoregma bambucicola* samples, Table S3: The details of OTUs distribution in all samples.
